# Visual Detection and Tracking System for a Spherical Amphibious Robot

**DOI:** 10.3390/s17040870

**Published:** 2017-04-15

**Authors:** Shuxiang Guo, Shaowu Pan, Liwei Shi, Ping Guo, Yanlin He, Kun Tang

**Affiliations:** 1 Key Laboratory of Convergence Medical Engineering System and Healthcare Technology, the Ministry of Industry and Information Technology, School of Life Science, Beijing Institute of Technology, Beijing 100081, China; guo@eng.kagawa-u.ac.jp (S.G.); panshaowu@bit.edu.cn (S.P.); guoping1962@hotmail.com (P.G.); heyanlin@bit.edu.cn (Y.H.); tangkun@bit.edu.cn (K.T.); 2 Faculty of Engineering, Kagawa University, 2217-20 Hayashicho, Takamatsu, Kagawa 761-0396, Japan

**Keywords:** spherical amphibious robot, Gaussian mixture model, moving target detection, system-on-chip (SoC), visual tracking

## Abstract

With the goal of supporting close-range observation tasks of a spherical amphibious robot, such as ecological observations and intelligent surveillance, a moving target detection and tracking system was designed and implemented in this study. Given the restrictions presented by the amphibious environment and the small-sized spherical amphibious robot, an industrial camera and vision algorithms using adaptive appearance models were adopted to construct the proposed system. To handle the problem of light scattering and absorption in the underwater environment, the multi-scale retinex with color restoration algorithm was used for image enhancement. Given the environmental disturbances in practical amphibious scenarios, the Gaussian mixture model was used to detect moving targets entering the field of view of the robot. A fast compressive tracker with a Kalman prediction mechanism was used to track the specified target. Considering the limited load space and the unique mechanical structure of the robot, the proposed vision system was fabricated with a low power system-on-chip using an asymmetric and heterogeneous computing architecture. Experimental results confirmed the validity and high efficiency of the proposed system. The design presented in this paper is able to meet future demands of spherical amphibious robots in biological monitoring and multi-robot cooperation.

## 1. Introduction

With the increased interest in ocean exploitation activities, amphibious robots have become essential tools for applications such as ecological observations and military reconnaissance in littoral regions [1,2]. Compared with legged, finned, and snakelike amphibious robots, spherical amphibious robots generate less noise and water disturbance, providing better stealth ability and bio-affinity [3,4]. Driven by pendulums [5], propellers [6] or rollers [7], most spherical amphibious robots are able to move flexibly in mud, snow or water with a zero turning radius. In 2012, our team proposed a novel spherical amphibious robot [8]. With a deformable mechanical structure, it walked on land with legs and swam in water with vectored propellers, which provided better overstepping ability and adaptability in littoral regions.

Moving target detection and tracking is a fundamental function for spherical amphibious robots to complete missions such as multi-robot formation, biological investigations, target localization, and navigation. The detection and tracking system recognizes an approaching target and then successively calculates its motion trajectory. Due to the restrictions presented by the environment, few sensors are suitable for detection and tracking applications of small-sized amphibious robots which have limited carrying capacities and battery power. As a fundamental telemetry device for most autonomous underwater vehicles (AUVs), acoustic sensors (e.g., side-scan sonar, single-beam sonars, and multi-beam sonars) can be effective over medium-to-long distances, however, most acoustic sensors are heavy and not suitable for observations at short distances (i.e., within 10 m) [9,10]. Optical and sonic tags broadcasting specific codes can be used to mark targets of interest (e.g., fish) for precise tracking over long distances [10,11]. However, this method is limited by the target size and provides few details on the surroundings or the target. Optical sensors, based on scanning light detection and ranging (LiDAR), laser-line scanning, structure light, and photometric stereo, have been used for underwater three-dimensional (3D) reconstruction [12]. However, most optical 3D reconstruction solutions require sophisticated optical structures and data processing devices, making it difficult to integrate such systems into a sub-meter-size spherical amphibious robot. With advantages in terms of weight, flexibility, and environmental adaptability, a visual detection and tracking system has been the key sensing equipment for small-scale spherical amphibious robots in executing close-range observation or inspection tasks in amphibious scenarios.

Although great progress has been achieved in the field of ground robotic vision, it still remains a challenging task to design a robotic detection and tracking system for spherical amphibious robots. First, image degradation is a major problem in underwater environments, which greatly impacts the performance of robotic visual systems. Second, interfering factors such as partial object occlusion, light variance, and pose changes are common in the potential application scenarios of amphibious robots, which may lead to detection or tracking failures. Third, most amphibious robots are small-sized and have relatively weak image processing power. Thus, both the visual algorithms and processing circuits must be carefully designed and optimized. As far as we know, most studies on robotic vision systems were conducted in terrestrial environments. Some visual detection and tracking systems have been designed for underwater robots or underwater surveillance networks, but few studies have involved amphibious robots.

Yahyaa et al. [13] proposed a visual detection and tracking system to guide an AUV towards its docking station. A tracker using color thresholding and morphological operations was designed to track artificial objects, but the robotic vision system could only recognize and track specific red light sources. Zhang et al. [14] presented a multi-target tracking system using multiple underwater cameras. An extended Kalman filter was initialized to track the moving targets. Speed up robust features (SURF) and random sample consensus (RANSAC) algorithms were used to match the target objects across the overlapping fields of view, but the cameras used were static and the visual system had poor real-time performance, which made it unsuitable for robotic applications. Chen et al. [15] proposed a novel 3D underwater tracking method in which haze concentration was used to estimate the distance between the target and the camera. However, this only provides the motion trends of underwater objects rather than precise measurements. Chuang et al. [16] proposed a robust multiple fish tracking system using Viterbi data association and low-frame-rate underwater stereo cameras. But it could only work in dark environments and provide a frame rate as low as 5 frame per second (fps). Chuang et al. [17] proposed a novel tracking algorithm on the basis of the deformable multiple kernels to track live fish in an open aquatic environment. Inspired by the deformable part model technique, the algorithm outperformed the recent tracking-by-detection algorithms for tracking one or multiple live fish in challenging underwater videos. But it could also provide a frame rate lower than 1 fps, which limited its applications in mobile robotic platforms.

In general, most existing robotic detection and tracking systems adopted vision algorithms using static or coarse appearance models [13,18], making them only capable of effectively processing specific targets such as fish and beacons under the specific scenes. There were some detection and tracking systems which adopted state-of-the-art visual algorithms and was capable of processing generic targets [16,17,19]. Given the real-time performance of these sophisticated algorithms, these systems have to be built upon high performance computers, making them only suitable for large-scale underwater robots or ground robots. Thus, existing solutions cannot be used directly in the small-sized amphibious spherical robot, which has limited load space and computational capabilities.

Focusing on the tasks of ecological observations and intelligent surveillance in littoral regions, a moving target detection and tracking system was proposed for our amphibious spherical robot in this study. Given the potential application scenarios of the robot, an industrial camera and vision algorithms using adaptive appearance models were used to construct the designed system. To handle the problem of light scattering and absorption in the underwater environment, the multi-scale retinex with color restoration (MSRCR) algorithm was used for image enhancement. In the detection stage, the amphibious spherical robot lurked in the surveyed region in hibernation mode and sensed the surroundings by capturing 320 × 240 color images at 15 fps. The Gaussian mixture model (GMM) was used to sense moving targets entering the robot’s view field. Once a moving target had been detected, the robot was woken up to the tracking stage. A fast compressive tracker (FCT) with a Kalman prediction mechanism was launched to locate the target position successively. Considering the limited load space and power resources of the robot, the designed visual detection and tracking system was implemented with a low-power system-on-chip (SoC). A novel asymmetric and heterogeneous computing architecture was used to ensure the real-time performance of the system. Experimental results confirmed that the proposed system was capable of detecting and tracking moving targets in various amphibious scenarios. In comparison with most relevant detection and tracking systems, the proposed system outperformed in terms of processing accuracy, environmental adaptability, real-time performance, and power consumption. It was able to meet future demands of the amphibious spherical robot in biological observation and multi-robot cooperation. The study in this paper provided a reference design to vision systems of small-sized amphibious robots.

The rest of this paper is organized as follows. An overview on our amphibious spherical robot and its vision application requirements is introduced in Section 2. The structure of the proposed vision system is presented in Section 3. Details of the underwater image enhancement subsystem and the detection-then-tracking subsystem are described in Section 4 and Section 5. Experimental results under various scenarios are reported in Section 6. Section 7 provides our conclusions and relevant follow-up research work.

## 2. Previous Work and Application Requirements

### 2.1. An Amphibious Spherical Robot

**Figure 1:**
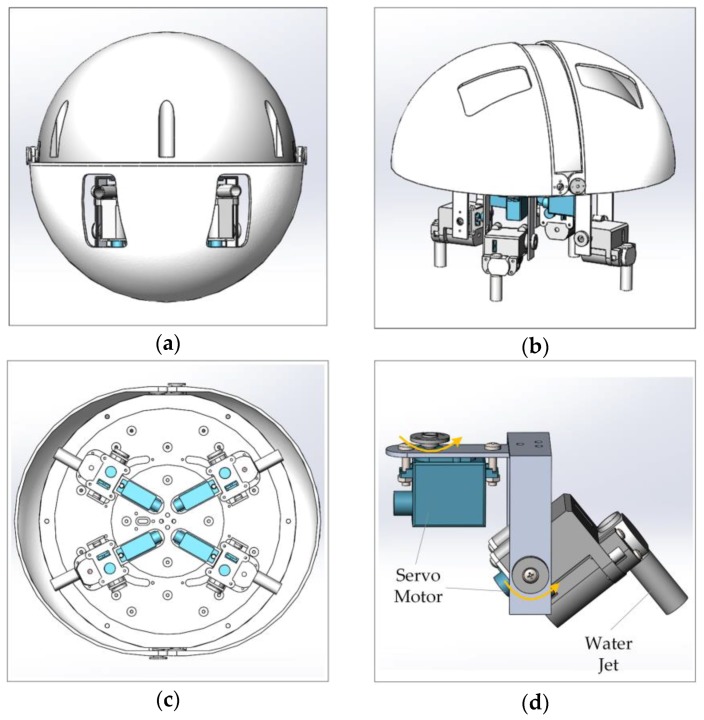
Mechanical structure of the amphibious spherical robot. (**a**) The amphibious spherical robot in the underwater mode; (**b**) The amphibious spherical robot in the land mode; (**c**) Bottom view of the amphibious spherical robot; and (**d**) The mechanical structure of a leg.

Figure 1 shows the amphibious spherical robot, which consisted of an enclosed hemisphere hull (diameter: 250 mm) and two openable quarter-sphere shells (diameter: 266 mm). Electronic devices and batteries were installed inside the hemispherical hull, which was waterproof and provided protection from collisions. Four legs, each of which was equipped with two servo motors and a water-jet motor, were installed symmetrically in the lower hemisphere of the robot. Driven by the two servo motors, the upper joint and lower joint of a leg were able to rotate around a vertical axis and a horizontal axis, respectively. The water-jet motor was fixed in the lower joint and could generate a vectored thrust in a specific direction in water. In underwater mode, the openable shells closed to form a ball shape, and the robot was propelled by vectored thrusts from four water jets. In land mode, the openable shells opened and the robot walked using the four legs.

**Figure 2:**
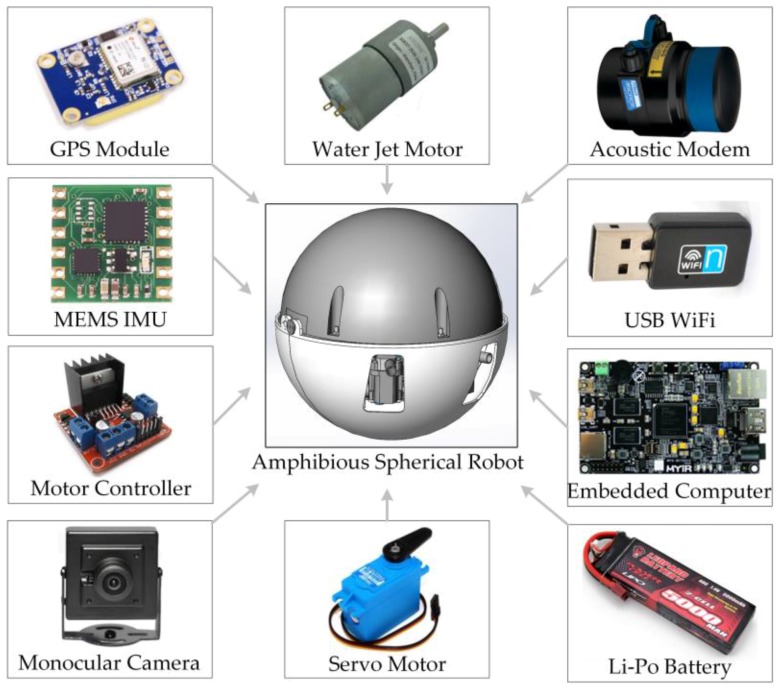
Major functional components of the amphibious spherical robot.

Restricted by the narrow load space and limited power resources of the small-scale robot, the robotic electronic system was fabricated around an embedded computer (Xilinx XC7Z045 SoC, 512 MB DDR3 memory, Linux 3.12.0), as shown in Figure 2. The robot was powered by a set of Li-Po batteries, with a total capacity of 24,000 mAh. Sensors including a global positioning system (GPS) module, an inertial measurement unit (IMU), and an industrial camera were used to achieve adaptive motion control of the robot [20]. An acoustic modem and a universal serial bus (USB) radio module were used for communication on land and in water, respectively [21].

### 2.2. Vision Application Requirements

Due to the special working environment, a vision method is the preferred solution to realize intelligent functions of the spherical robot in amphibious scenarios. Compared with intelligent automobiles and large-scale AUVs, the amphibious spherical robot has higher requirements for the robotic vision system in terms of robustness and efficiency.

First, due to the uneven illumination and the optical properties of water, the captured image may be blurred and dusky. Thus, image pre-processing is essential to enhance visibility before implementing vision algorithms. Second, many interfering factors in amphibious environments, including swaying aquatic plants, suspended organic particles, illumination changes, and a cluttered background, may mislead the detector and the tracker. Thus, the robustness and precision of the adopted computer vision algorithms should be acceptable to meet the requirements of robotic applications. Third, the robot has a limited velocity and cruising ability. Thus, the captured image should be processed in real time to avoid missing a target or omitting information. Moreover, considering the narrow enclosed load space of the spherical robot, the hardware platform of the robotic vision system should be highly efficient to reduce power consumption and heat dissipation issues. Furthermore, the implementation of the adopted vision algorithms should be optimized carefully in accordance with characteristics of the hardware platform.

In 2015, a prototype moving target detection system was designed and constructed for the amphibious spherical robot [22]. A single Gaussian background model (GBM) was adopted to sense moving objects getting close to the robot and the heterogeneous computing technology was used to enhance the real-time performance. However, the prototype system did not perform well in practicality experiments because the illumination problem and the interfering factors in amphibious environments were not taken into consideration yet. Moreover, due to the principle of the adopted algorithm, the detection system could work normally only when the robot was static, which was not the case. Besides, the coarse system architecture led to a slower respond speed of the control system, which affected the performance of the robot.

## 3. Visual Detection and Tracking System

### 3.1. Workflow of the System

Benefiting from its ball shape, the amphibious spherical robot generated fewer disturbances to the surroundings, which is meaningful in ecological and military applications [23]. Moreover, the symmetric mechanical structure contributed to the stable and flexible motion characteristics of the robot, making it a platform suitable for amphibious data acquisition. However, the compact size and the spherical shape of the robot also resulted in a limited cruising speed and a short continuous operating time. Thus, it was unable to complete search or investigation tasks over a large region. Indeed, it was more appropriate to use the robot as an intelligent and movable monitoring node for close-range ecological observations or security surveillance.

**Figure 3:**
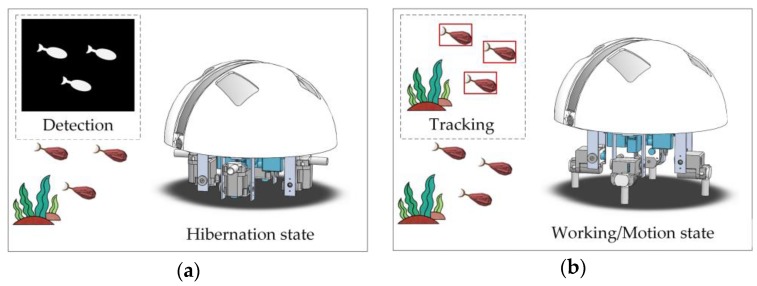
Application scenario of the visual detection and tracking system. (**a**) The moving target detection stage; and (**b**) The visual tracking stage.

A potential application scenario is shown in Figure 3. The working process was divided into the moving target detection stage and the visual tracking stage. In the moving target detection stage, the small-scale robot lurked in the survey region in hibernation mode. Most of its functional units, including motors, the data recording subsystem, and the acoustic modem, were shut down to avoid exhausting the batteries and storage resources too early. Enhanced images of surroundings were entered into the visual detection subsystem to search for moving targets, such as fish and swimmers. Once a moving object entered the view field of the robot and was marked as a target, the robot would be activated and switched to visual tracking mode. In the visual tracking stage, a visual tracker was launched to track the specified target. The tracking results were then used to guide the movement and follow-up operations of the robot.

### 3.2. Structure of the System

**Figure 4:**
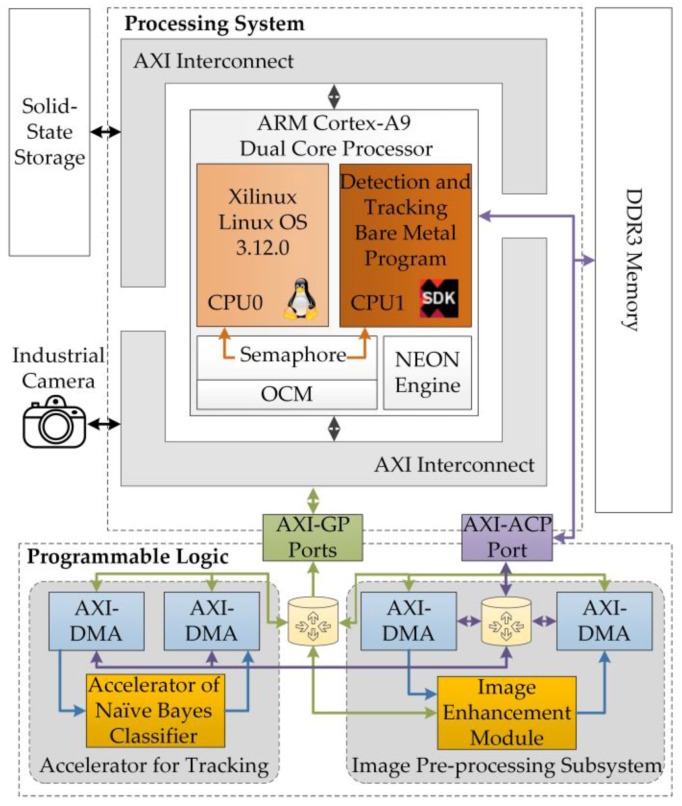
Hardware structure of the visual detection and tracking system.

The entire visual detection and tracking systems were integrated on a Xilinx XC7Z045 SoC, as shown in Figure 4. As the center of the robotic electronic system, the SoC consists of the processing system (PS), which is centered on a dual-core ARM processor, and the programmable logic (PL), which is equivalent to a field-programmable gate array (FPGA) [24]. The PL served as a customized peripheral of the PS and communicated with programs running on the PS through advanced extendable interface (AXI) ports.

To ensure balance between the power consumption of the electronic system and the real-time performance of the robotic vision system, an asymmetric and heterogeneous computing architecture was used to develop the visual detection and tracking system. The CPU0 ran the Linux operating system (OS), which provided a multi-task platform for basic robotic functions, such as motion control and battery management. The CPU1 ran bare-metal programs for real-time detection and tracking. Customized accelerators deployed on the PL assisted the bare-metal programs to ensure real-time performance. The application programs running in the Linux OS communicated with the bare-metal programs through a shared on-chip memory. The 320 × 240 color images to be processed were captured by an industrial camera mounted on a USB port of the SoC. To address the problem of image degradation, an image-enhancement module using the MSRCR algorithm was implemented on the PL for real-time image pre-processing. A customized accelerator for the naïve Bayes classifier was deployed on the PL to speed up the bare-metal visual tracking program. Two pairs of direct memory access (DMA) channels were used to read unprocessed data from the PS and then transmit the processed results back to the bare-metal programs.

## 4. Image Pre-Processing Subsystem

### 4.1. Principle of the Image Pre-Processing Algorithm

Due to the short sensing range and the image degradation problem, cameras have not been at the center of attention as underwater robotic sensors. The degradation of underwater images is caused primarily by multiple factors including light attenuation, light scattering, and suspended organic particles [18]. Existing underwater enhancement algorithms can be divided loosely into four classes. The time domain algorithms and the frequency domain algorithms enhance image quality using ‘classical’ digital image processing techniques such as histogram equalization and homomorphic filtering. The physics-based algorithms build an optical model of underwater imaging devices and recover the image visibility using optical components [25,26]. The algorithms based on the theory of color constancy were inspired by the human vision system and seek to depress image degradation caused by illumination factors [27,28]. Among these algorithms, the MSRCR algorithm provides a good processing effect by taking advantage of multi-scale analysis and color recovery.

**Figure 5:**
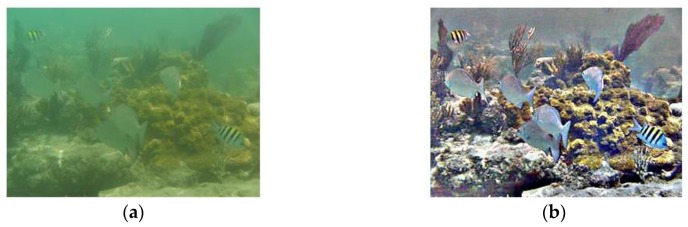

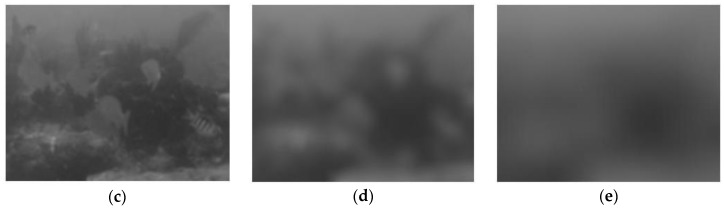
Diagram of the image pre-processing algorithm. (**a**) The original 320 × 240 image; (**b**) The enhanced image; (**c**) Estimate of *L*(*x*, *y*) using the 5 × 5 Gaussian filter; (**d**) Estimate of *L*(*x*, *y*) using the 24 × 24 Gaussian filter; and (**e**) Estimate of *L*(*x*, *y*) using the 48 × 48 Gaussian filter.

The MSRCR algorithm was inspired by the model of lightness and color perception of human vision. The retinex theory holds that the image projected onto the retina *I*(*x*, *y*) is determined by the illumination component *L*(*x*, *y*) and the relative reflectance component *R*(*x*, *y*):

*I*(*x*, *y*) = *L*(*x*, *y*) · *R*(*x*, *y*), (1)

where *x* and *y* represent the coordinate of an image. Thus, the negative influence of light scattering and absorption can be excluded by estimating *L*(*x*, *y*). As shown in Figure 5c, an estimate of *L*(*x*, *y*) can be acquired using a Gaussian low-pass filter:

(2) $$\hat{L}\left(x,y\right)=I(x,y)*k\exp(-\frac{x^{2}+y^{2}}{\sigma^{2}}),$$

where *σ* represents the scale of the Gaussian filter. Then, the relative reflectance component *R*(*x*, *y*) can be represented as

(3) R(x,y)=log(I(x,y))−log(F(x,y)∗I(x,y)),

where *F*(*x*, *y*) represents the Gaussian filter.

**Figure 6:**
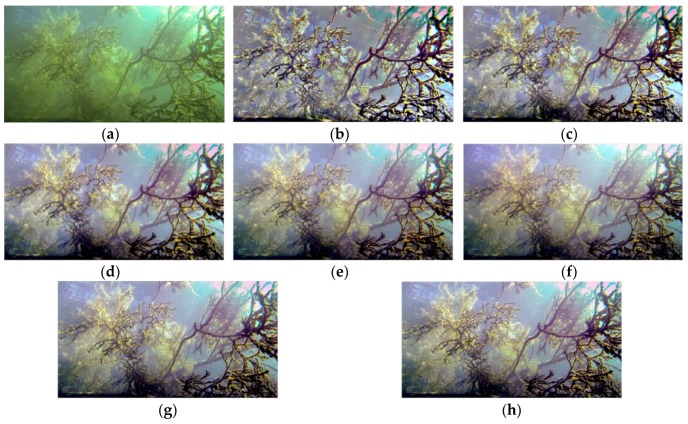
Comparison of the multi-scale retinex with color restoration (MSRCR) algorithm with different parameters. (**a**) The original 852 × 480 image; (**b**) Enhanced image (*n*_S_ = 1, *σ*_max_ = 50); (**c**) Enhanced image (*n*_S_ = 1, *σ*_max_ = 100); (**d**) Enhanced image (*n*_S_ = 1, *σ*_max_ = 200); (**e**) Enhanced image (*n*_S_ = 1, *σ*_max_ = 300); (**f**) Enhanced image (*n*_S_ = 2, *σ*_max_ = 300); (**g**) Enhanced image (*n*_S_ = 3, *σ*_max_ = 300); and (**h**) Enhanced image (*n*_S_ = 4, *σ*_max_ = 300).

The value of *σ* is important for the retinex algorithm, especially for an image with non-uniform background illumination. A small *σ* works better on dark regions of the image, and a large *σ* leads to better color constancy, as shown in Figure 6b–e. To make use of the strength of multi-scale synthesis, the MSRCR algorithm combining multiple scales is commonly used with linear weighting:

(4) $$R\left(x,y\right)=\log(I(x,y))-\sum_{i=1}^{n_{S}}w_{i}\log(F_{i}(x,y)*I(x,y)),$$

where *n*_S_ represents the number of adopted filter scales. Both the details and the color constancy of the processed image can be ensured using a *n*_S_ > 3, as shown in Figure 6f–h. A larger *n*_S_ may lead to better algorithm performance and higher computational needs. Given the characteristics of the robot and the size of images to be processed, the proposed system adopted three scales (*σ*_1_ = 5, *σ*_2_ = 24, and *σ*_3_ = 48), which balanced the image contrast, color constancy, and computational efficiency.

### 4.2. Image Pre-Processing Subsystem

**Figure 7:**
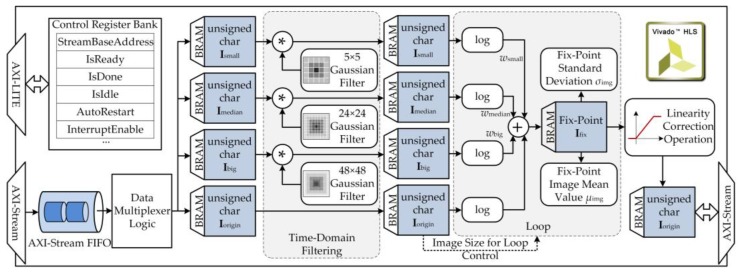
Diagram of the image pre-processing subsystem.

The MSRCR algorithm involves large amounts of multiplication operations, which are time-consuming. To ensure real-time performance of the proposed robotic vision system, a customized IP core was designed to implement the MSRCR algorithm using high-level synthesis (HLS) tools. A 320 × 240 24-bit color image was read serially from the DMA channel into the IP core through an AXI-Stream port, as shown in Figure 7. The color image was converted to an 8-bit gray image and then buffered into a slice of block RAM (BRAM). Next, three convolution operations were executed in parallel. Then, logarithmic transformations were carried out serially over the calculated $\hat{L}\left(x,\;y\right)$. Finally, the enhanced image *R*(*x*, *y*) was sent out through an AXI-Stream port after a linear color correction operation.

The low-pass filtering and standard deviation calculation functions were designed with C++, referring to their counterparts in the OpenCV library. In the convolution operation, the input image was extended at the boundary with the edge pixel not duplicated. Because the quality of synthesis results provided by HLS tools are less than ideal, it was essential to conduct design optimization. To reduce resource consumption of the PL, the filter parameters were represented in the accelerator by fixed-point approximations. The synthesis report showed that the operation time of the designed IP core was ~48.0 ms, which was 3.7 times faster than the software implementation on the PS.

## 5. Detection and Tracking Subsystem

### 5.1. Moving Target Detection Subsystem

As mentioned in Section 3, the robot sensed moving objects entering its observation field and then specified an eligible one as a target to be tracked. A common method for moving target detection is using background subtraction or motion detection algorithms that have been used successfully in intelligent surveillance systems. State-of-the-art background subtraction algorithms, such as most reliable background mode (MRBM) and effect components description (ECD) demand large amounts of memory and/or computing time [29], making them unsuitable for use in the amphibious spherical robot. Moreover, ‘classical’ algorithms, such as the frame difference algorithm and the weighted moving mean algorithm, may easily be misled by interfering factors, including swaying aquatic plants and suspended organic particles in practical applications [30].

Thus, the adaptive Gaussian mixture model for foreground detection proposed by Kaewtrakulpong et al. [31,32], which has good detection precision and is able to neglect noises caused by background jitter, was adopted in the proposed robotic vision system. An overview on the principles of the adopted moving target detection algorithm is shown in Algorithm 1. Each pixel of the input image was modeled with a mixture of K Gaussian distributions:

*R*(*x*, *y*) ~ *w_x,y,k_ N*(*μ_x,y,k_, σ_x,y,k_*), (5)

where *μ_x,y,k_*, *σ_x,y,k_*, and *ω_x,y,k_* are parameters of the *k*th Gaussian component. The K Gaussian distributions are ordered based on the fitness value *ω_x,y,k_*/*σ_x,y,k_*. The top B distributions constituted the background model where B was defined as:

(6) $$B=\arg\underset{b}{\min}(\sum_{k=1}^{b}w_{x,y,k}>T).$$

If an input pixel was less than *d* standard deviations away from any of the distribution of the background model, it was regarded as belonging to the background scene. Otherwise, it was regarded as part of the potential moving target. Algorithm parameters were updated with the learning rate α to adapt to environmental changes. The detected foreground image was processed with erode and dilate operations to filter noise. A moving object larger than *Area*_Thresh_ would be specified as the target to be tracked in the following processes.

**Algorithm 1.** Gaussian mixture model-based moving target detection**input:** the enhanced image *R_x_*_, *y*_ and parameters of Gaussian mixture model *μ_x,y,k_*, *σ_x,y,k_* and *ω_x,y,k_*, where *x* ∈ [1,Width], *y* ∈ [1,Height], *k* ∈ [1,K]**output:** the foreground image *F_x_*_, *y*_, where *x* ∈ [1,Width], *y* ∈ [1,Height]**procedure** GaussianMixtureModelDetection(**R**, **μ**, **σ**, **w**)  **Step #1** Initialize the parameters of Gaussian mixture model    *μ_x,y,k_*←rand(), *σ_x,y,k_*←*σ*_0_, *ω_x,y,k_*←1/K  **Step #2** Try to match the Gaussian mixture model with the *n*-th image    **for**
*k* = 1 to K **do**      **if**
*R_x,y,n_*
−
*μ_x,y,k_* < *d*·*σ_x,y,k_*
**then**      *match_k_* = 1      *ω_x,y,k_* = (1 *− α*)·*ω_x,y,k_ + α*      *μ_x,y,k_* = (1 *− α*/*ω_x,y,k_*)·*μ_x,y,k_ + α*/*ω_x,y,k_*·*R_x,y,n_*      *σ_x,y,k_* = $\sqrt{(1-\alpha /w_{x,y,k})\cdot \sigma_{{}_{x,y,k}}^{2}+\alpha /w_{x,y,k}\cdot {\left(R_{x,y,k}-\;\mu_{x,y,k}\right)}^{2}}$      **else**      *ω_x,y,k_* = (1 *− α*)·*ω_x,y,k_*      **end if**    **end**    **Step #3** Normalize the weight *ω_x,y,k_* and sort the model with *ω_x,y,k_*/σ*_x,y,k_*    **Step #4** Reinitialize the model with minimum weight if there is no matched model,      **if**
$\sum_{k=1}^{K}match_{k}=0$
**then**      *μ_x,y,_*_0_ = *pixel_x,y,n_*      *σ_x,y,k_* = *σ*_0_      **end if**    **Step #5**      **for**
*k* = 1 to K **do**      **if**
*ω_x,y,k_* > T **and**
*R_x,y,n_*
−
*μ_x,y,k_* < *d*·*σ_x,y,k_*
**then**        *F_x, y_* = 0        **break**      **else**        *F_x, y_* = 255      **end if**      **end**  **Step #6** Execute 3 × 3 erode and dilate operations over *R*(*x*, *y*)  **Step #7** Execute connected region analysis and list potential moving target  **Step #8** Specify the object larger than *Area*_Thresh_ as the target**end procedure**

The designed moving target detection subsystem was implemented as a bare-metal program running on CPU1. The number of Gaussian distributions was set to 4. The NEON engine was used to optimize the floating-point arithmetic of the bare-metal program, which increased the detection rate from 7.4 fps (135.16 ms/f) to 19.7 fps (50.8 ms/f). If a moving object was detected, the detector would inform CPU0 by writing the coordinate of the target to a specific memory location. Then, the tracking subsystem would be launched to handle the target.

### 5.2. Visual Tracking Subsystem

The major task of the visual tracking subsystem was successively marking the position of the specified target for robotic applications. As an active research field in the area of computer vision, visual tracking is the basis of high-level robotic functions, such as automatic navigation, visual servoing, and human–machine interactions [33,34]. Many state-of-the-art tracking algorithms, built on tracking-by-detection [35], correlation filtering [36], and convolutional neural networks [37], have been proposed in recent years. However, it is still challenging to ensure both high tracking precision and real-time performance, limiting their use in small-scale mobile robot platforms.

**Figure 8:**
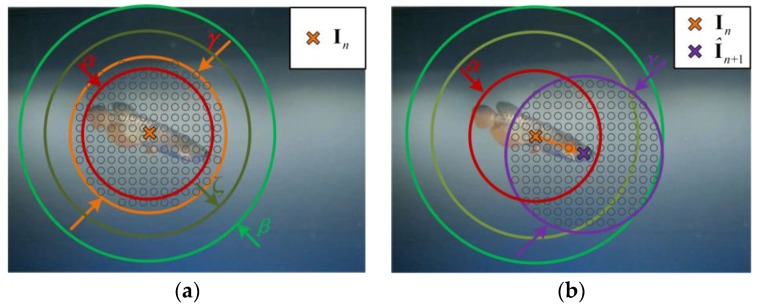
Principle of the visual tracking algorithm. (**a**) Original fast compressive tracker (FCT) algorithm; and (**b**) Improved FCT algorithm.

The fast compressive tracking (FCT) algorithm was selected in the visual tracking subsystem as it offers the advantages of effectiveness and efficiency [38]. As a tracking-by-detection algorithm with online learning mechanisms, the FCT algorithm contains a training stage and a detection stage. In the training stage at the *n*th frame, the tracker densely crops positive samples **S**_pos_ and negative samples **S**_neg_ around the current target position **I***_n_*, as shown in Figure 8a:

(7) $$S_{\mathrm{pos}}=\text{\{}s|\left\|s-I_{n}\right\|<\alpha \text{\}},$$

(8) $$S_{\mathrm{neg}}=\text{\{}s|\zeta <\left\|s-I_{n}\right\|<\beta \text{\}},$$

where *α* < *ζ* < *β*. Then, random Haar-like features of samples were extracted using a static sparse matrix. Affected by the optical properties of water, it is not easy to extract local invariant image features in underwater vision applications. Thus, a global feature like the random Haar-like features was more effective in the designed system. After that, a naïve Bayes classifier was trained using feature vectors of the samples. In the detection stage at the (*n* + 1)th frame, candidate samples **S**_can_ were densely cropped around **I***_n_* using a coarse-to-fine mechanism:

(9) $$S_{\mathrm{can}}=\text{\{}s|\left\|s-I_{n}\right\|<\gamma \text{\}}$$

The candidate with the maximum classifier response was selected as the current target I*_n_*_+1_. Regarding the vision application of the amphibious spherical robot, there are two potential problems that may affect the performance of the FCT algorithm. One is that the FCT algorithm is not good at maneuvering target tracking due to its sampling mechanism. Moreover, the tracker may lose the target in this robotic vision system with its relatively low frame rate. To address this, a second-order Kalman filter was used to predict the target position at the (*n* + 1)th frame in the detection stage.

(10) $$\left\{ \begin{array}{l} X_{n+1}=\Phi X_{n}+\beta W_{n}\\ {\hat{I}}_{n+1}=HX_{n+1}+\alpha V_{n}\\ \Phi =\left(\begin{array}{cccccc} 1 & 0 & \Delta t & 0 & \Delta t^{2}/2 & 0\\ 0 & 1 & 0 & \Delta t & 0 & \Delta t^{2}/2\\ 0 & 0 & 1 & 0 & \Delta t & 0\\ 0 & 0 & 0 & 1 & 0 & \Delta t\\ 0 & 0 & 0 & 0 & 1 & 0\\ 0 & 0 & 0 & 0 & 0 & 1 \end{array}\right)\\ H=\left(\begin{array}{cccccc} 1 & 0 & 0 & 0 & 0 & 0\\ 0 & 1 & 0 & 0 & 0 & 0 \end{array}\right)\\ X_{n}={\left(x_{n},y_{n},v_{x,n},v_{y,n},a_{x,n},a_{y,n}\right)}^{T}\\ {\hat{I}}_{n}={\left({\hat{x}}_{n},{\hat{y}}_{n}\right)}^{T} \end{array}\right.,$$

where (*x_n_*, *y_n_*), (*v_x_*_,*n*_, *v_y_*_,*n*_), and (*a_x_*_,*n*_, *a_y_*_,*n*_) are the position, the velocity, and the acceleration of the target in the *n*th frame, respectively. The candidate samples **S**_can_ would be sampled around the estimate position ${\hat{I}}_{n+1}$ rather than **I***_n_*, as shown in Figure 8b:

(11) $$S_{\mathrm{can}}=\text{\{}s|\left\|s-{\hat{I}}_{n+1}\right\|<\gamma \text{\}}$$

Because most moving objects in water have a stable trajectory, the improved tracker was able to adapt to the motion by predefining appropriate parameters.

Another problem was that the floating-point arithmetic processes of the FCT algorithm, especially the naïve Bayes classification process, could not be processed efficiently by CPU1:

(12) $$H_{\mathrm{pos},i}(v)=\frac{\exp(-{\left(v_{i}-\mu_{\mathrm{pos},i}\right)}^{2}/\left(2\sigma_{\mathrm{pos},i}^{2}+10^{-30}\right)}{\mu_{\mathrm{pos},i}+10^{-30}},$$

(13) $$H_{\mathrm{neg},i}(v)=\frac{\exp(-{\left(v_{i}-\mu_{\mathrm{neg},i}\right)}^{2}/\left(2\sigma_{\mathrm{neg},i}^{2}+10^{-30}\right)}{\sigma_{\mathrm{neg},i}+10^{-30}},$$

(14) $$H(v)=\sum_{i=1}^{m}\left(\log(H_{\mathrm{pos},i}(v)+10^{-30}\right)-\log(H_{\mathrm{neg},i}(v)+10^{-30})),$$

where $v\in {\mathbb{R}}^{m}$ represents the feature vector of a candidate sample and **μ**_pos_, **μ**_pos_, **σ**_pos_, and **σ**_neg_ represent classifier parameters. As described by Equations (12)–(14), the naïve Bayes classification process primarily concerns the exponent and logarithm, which are equivalent to iterative multiplication operations. Thus, a customized accelerator may perform better than a general-purpose accelerator in speeding up these calculations.

**Figure 9:**
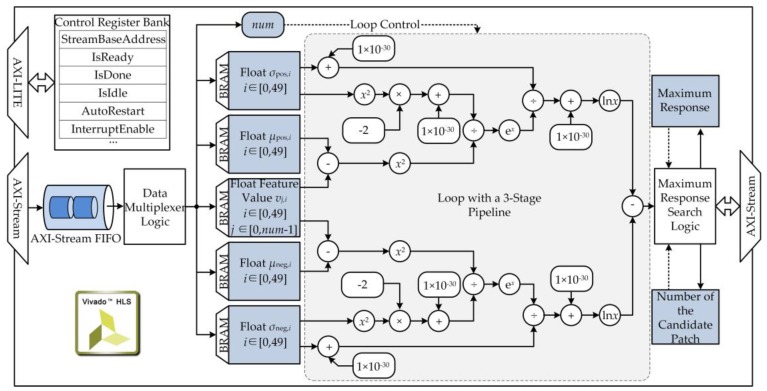
Diagram of the accelerator of naïve Bayes classifier for the tracking subsystem.

A customized accelerator of the naïve Bayes classifier was designed and implemented on the PL section using HLS tools, as shown in Figure 9. The sampled feature vectors and classifier parameters were read from the bare-metal program running on the PS through a DMA channel and then buffered into BRAM slices. Then, a three-stage pipeline was designed to complete the classifier response calculation loop in parallel. Finally, the maximum response and the number of the candidate sample were found and sent back to the PS through a DMA channel. Using the customized accelerator, the average processing rate of the heterogeneous tracking subsystem was 56.3 fps (17.8 ms/f), which was 4.1 times faster than the software implementation on the PS (13.6 fps or 73.5 ms/f).

## 6. Experimental Results

**Figure 10:**
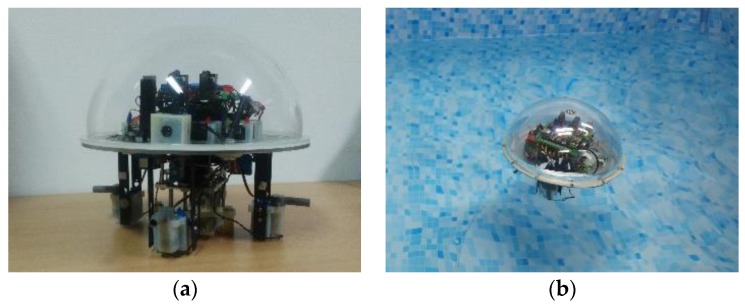
Picture of the proposed robotic vision system. (**a**) Installation of the vision system; and (**b**) Picture of the robot in working state.

The improved version of the amphibious spherical robot is shown in Figure 10. A core board carrying the Xilinx SoC (XC7Z045) and an industrial CMOS camera were used to assemble the proposed detection and tracking system. Then, the embedded robotic vision system was sealed in the upper hemisphere of the spherical robot using a transparent shell. To confirm the validation of the proposed robotic vision system, two phases of experiments were conducted to test its detection and tracking precision, real-time performance, and power consumption.

(1) In the parametric test phase, an Agilent 34410A multimeter, controlled by C# programs, was used to evaluate the average power consumption of the proposed system by continuously measuring the current and voltage values. The power consumption of the robot in idle mode was regarded as the baseline. Test results showed that the dynamic power consumption of the proposed system was as low as 4.57 W, which was able to provide a continuous working time more than 2.5 h. To test the process rate of the proposed system, eight 320 × 240 image sequences (*CarDark*, *Trellis*, *David*, *Couple*, *Fish*, *Dog1*, *Sylvester*, and *ClifBar*) of Visual Tracker Benchmark [39,40] were entered into the system using debugging tools. The run time of each subsystem were measured using a hardware counter deployed on the PL section, respectively. Test results indicated that the system was able to provide an average pre-processing rate of 20.8 fps, an average detection rate of 19.7 fps, and an average tracking rate of 56.3 fps. Thus, it was able to process images captured by the industrial camera in amphibious scenarios in real time. As shown in Table 1, the proposed system had advantages in real-time performance, power consumption, size and weight, which could fully meet the application requirements of the amphibious spherical robot.

(2) In the detection and tracking test phase, four images sequences captured in various amphibious scenarios were used to evaluate functional performance of the system. The ground truth of detection and tracking were annotated manually. The proposed detection subsystem was compared with the GBM-based detection algorithm. The proposed tracking subsystem was compared with three state-of-the-art discriminative tracking algorithms (CT [38], WMIL [35], and HOG-SVM [43]) and five classical tracking algorithms (TemplateMatch, MeanShift, VarianceRatio, PeakDifference and RatioShift [44]) which were widely used in robotics. Four metrics were used to evaluate the functional performance of the detection and tracking system. The first metric is the percentage of wrong classifications (*PWC*) of the detection process, defined as:

(15) PWC=(FP+FN)/(TP+TN+FP+FN)×100,

where *TP*, *TN*, *FP*, and *FN* represent the number of true positive, true negative, false positive, and false negative pixels, respectively. The second metric is the precision (*Pr*) of the detection process, defined as:

(16) Pr=TP/(TP+FP).

The third metric is the success rate (*SR*) of the tracking process, defined as:

(17) $$SR=\frac{area(ROI_{T}\cap ROI_{G})}{area(ROI_{T}\cup ROI_{G})},$$

where *ROI*_T_ is the tracked bounding box, *ROI*_G_ is the ground truth bounding box, and *area*(·) denotes the number of pixels in the region. If the *score* is larger than the given threshold (0.5 in this study) in a frame, it counts as a success. The fourth metric is the center location error (*CLE*), which is the Euclidean distance between the central points of the tracked bounding box and the ground truth bounding box.

**Figure 11:**
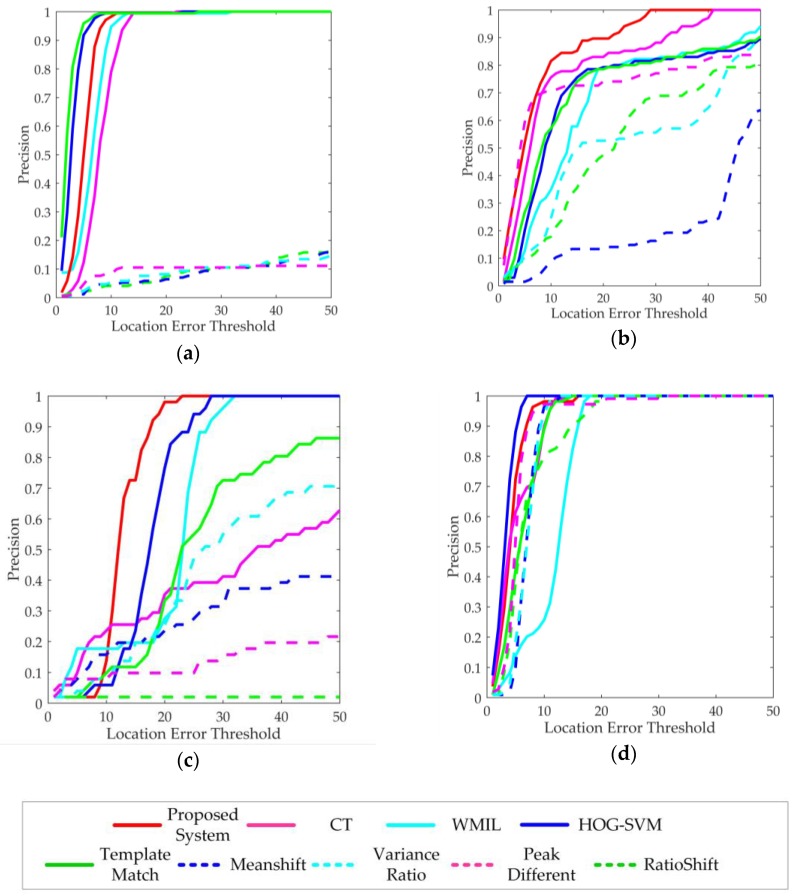
Precision plot of tracking for test image sequences. (**a**) Precision plot for Sequence 1; (**b**) Precision plot for Sequence 2; (**c**) Precision plot for Sequence 3; and (**d**) Precision plot for Sequence 4.

As shown in Table 2, the proposed detection subsystem provided the 47.7% lower *PWC* and the 17.2% higher *Pr* on average than the GBM-based detection algorithm. Thus, the proposed detection subsystem was more robust to environmental disturbances. As shown in Table 3 and Figure 11, the proposed tracking subsystem outperformed other tracking algorithms in terms of the *SR* and the *CLE*. The five classical tracking algorithms were not able to steadily track underwater targets in the tests of Sequence 1, Sequence 2, and Sequence 3 because they adopted static or coarse appearance models. The CT and WMIL algorithms adopted adaptive appearance models, but they were lack of effective motion prediction or dynamic update mechanisms. Thus, they did not perform well in some scenarios due to the drift problem. The HOG-SVM algorithm adopted an effective feature extractor and a strong classifier. Thus, it performed better than the proposed tracking subsystem in the tests of Sequence 1 and Sequence 4. But it was a non-real-time tracking algorithm and could only provide a processing rate as low as 2.7 fps. Thus, it was not suitable for the applications of the amphibious spherical robot. In general, the discriminative tracking algorithms using adaptive appearance models performed better than the classical tracking algorithms, especially in the underwater environments.

**Figure 12:**
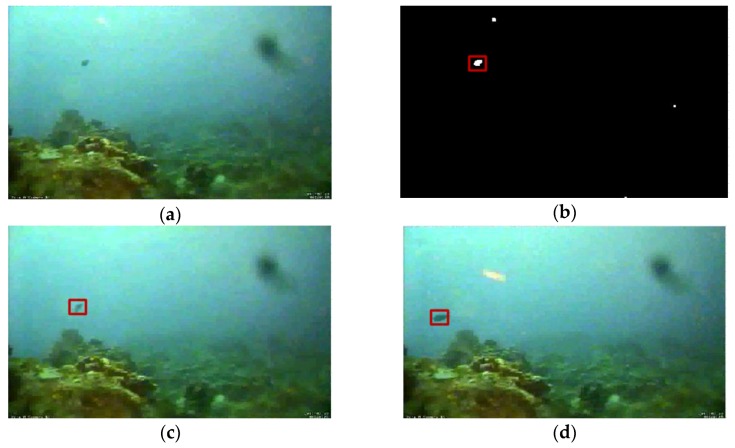
Experimental results of Sequence 1. (**a**) Image collected from the underwater observatory; (**b**) Detection result; (**c**) Tracking result; and (**d**) Tracking result.

**Figure 13:**
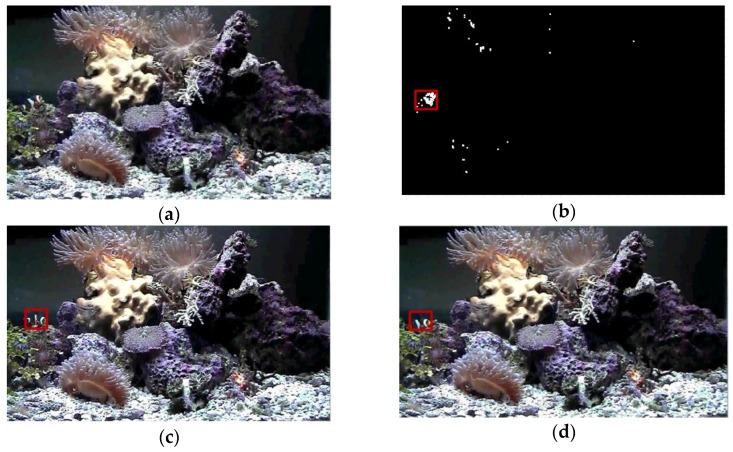
Experimental results of Sequence 2. (**a**) Image collected from the underwater observatory; (**b**) Detection result; (**c**) Tracking result; and (**d**) Tracking result.

As shown in Figure 12 and Figure 13, two underwater videos of fishes provided by the Fish4Knowledge project [19] were used to evaluate the performance of the proposed system towards underwater targets. Sequence 1 was collected from the underwater observatory at Orchild Island, Taiwan. A distant moving fish with a stable motion trajectory was selected as the target. Due to the light scattering and absorption effect of ocean water, the captured underwater images were blurry, and the appearance characteristics of the target were not significant. In the test of Sequence 1, the proposed system was able to detect and then track the small moving fish with high accuracy. Most trackers without image pre-processing successively lost the target because the appearance characteristics were not so significant. Test results of Sequence 1 demonstrated that the proposed system was capable of detecting and tracking practical target in the undersea environment.

Sequence 2 was collected from the underwater observatory at National Museum of Marine Biology and Aquarium, Taiwan. A tropical fish moving randomly was selected as the target. The captured images were clear, and the target had obvious texture features. However, the ever-changing motion trajectory of the target and the swaying corals in the background would mislead the robotic vision system. The disturbance of swaying corals in Sequence 2 was neglected in the detection process by using the GMM-based method, ensuring the correct detection of the tropical fish. The GBM-based method got disturbed and provided higher error rate. However, because the proposed tracking subsystem as well as the three discriminative tracking algorithms do not have scale invariant and affine invariance properties, the visual trackers finally lost the target when the fish changed poses. The five classical tracking algorithms lost the target soon because the disturbances caused by similar objects in the background. Test results of Sequence 2 verified that the proposed system could provide relatively accurate detection and tracking results when working in the complex and cluttered underwater environment.

**Figure 14:**
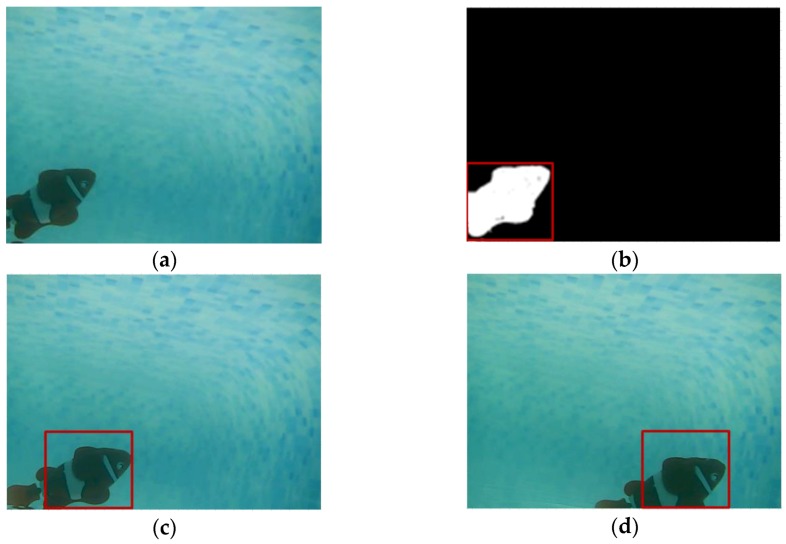
Experimental results of Sequence 3. (**a**) Image captured by the robot; (**b**) Detection result; (**c**) Tracking result; and (**d**) Tracking result.

**Figure 15:**
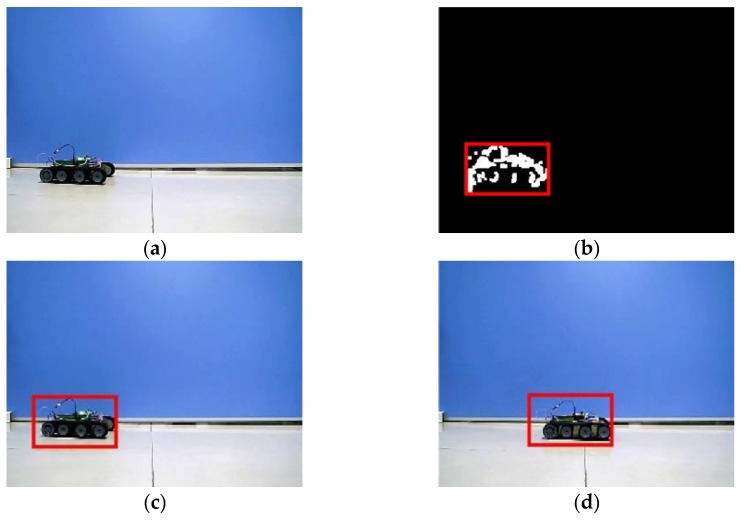
Experimental results of Sequence 4. (**a**) Image captured by the robot; (**b**) Detection result; (**c**) Tracking result; and (**d**) Tracking result.

Two videos captured by the amphibious spherical robot in underwater and terrestrial environments were used for evaluation, as shown in Figure 14 and Figure 15. Sequence 3 was collected from the amphibious spherical robot in a tank. A small toy fish swimming fast was adopted as the moving target. The image quality of Sequence 3 was better than that of Sequence 1. But the robotic platform rocked slowly with the water in a practical underwater scenario, which would present difficulties for the detection and tracking process. In the test of Sequence 3, the detection precision of the proposed system was acceptable even though the robotic platform was not so steady. Because the robot had a small view field and a low frame rate, the fish model swam at a relatively fast speed in the video. By predefining appropriate parameters for the Kalman filter, the proposed system could stably track the fish after detecting it. And the drift problem occurred when using the original CT algorithm, which resulted in worse tracking performance. The five classical trackers failed in the tracking process because they cannot adapt to the ever-changing target. Test results of Sequence 3 verified that the proposed system was capable of detecting and tracking the target object moving at fast speed and it could meet the application requirements of the amphibious target tracking in underwater environments.

Sequence 4 was collected from the amphibious spherical robot in the laboratory environment. A small tracked robot moving at a low speed was adopted as the moving target. The robotic platform was relatively stable, and the image quality was good. But the appearance characteristics of the target slowly changed, which might lead to the drift problem in the tracking process. In the test of Sequence 4, the detection results were not so good because the motion speed of the small car was slow. However, the target region of the small car was recognized correctly, and the GMM-based detector provided better *PWC* and *Pr* than the GBM-based detector. Except that the WMIL tracker encountered the drift problem, all the trackers were capable of successively tracking the specified target which remained nearly unchanged. That demonstrated that visual tracking in underwater environments is a much more challenge work than that in terrestrial environments from another side. Because most studies on robotic vision were conducted on land, which might be not suitable for underwater applications, optimizations were essential in the design of visual detection and tracking system for amphibious spherical robots. Test results of Sequence 4 verified that the proposed system was able to steadily detect and track the target object on land. And it could meet the application requirements of the amphibious in terrestrial environments.

## 7. Conclusions and Future Work

To meet the practical application requirements of the spherical amphibious robot in ecological observations and intelligent surveillance tasks, an embedded detection and tracking system was designed and implemented. To address the image degradation problem in underwater scenarios, captured images were pre-processed with the MSRCR algorithm to reduce the effects of light absorption and scattering. Then, the Gaussian mixture model was used to detect moving targets entering the robot’s view field. The marked target was tracked successively using a FCT tracker with a Kalman prediction mechanism. Using these algorithms with online learning mechanisms, the designed detection and tracking subsystems were able to resist disturbances, such as the swaying aquatic plants in the detection stage and the fast motion of a target in the tracking stage. Considering the unique mechanical structure and limited load space of the robot, the whole vision system was integrated into a low-power SoC using an asymmetric and heterogeneous computing architecture. Evaluation experiments confirmed the validation and efficiency of the proposed system. The proposed system was capable of precisely detecting and tracking various target objects in both underwater and terrestrial environments in real time. With the features of low power consumption, high-real-time performance, and good environmental adaptability, it was able to meet the potential demands of the small-sized spherical amphibious robot in multi-robot cooperation and multi-target tracking tasks. As far as we know, it was the first practical visual detection and tracking system towards generic targets for small-sized amphibious robots. In comparison with most relevant studies, the proposed system provided higher detection and tracking accuracy by implementing adaptive visual algorithms and introducing improvement methods. Built upon a heterogeneous embedded system, it could fit in well with the characteristics of small-sized amphibious and underwater robots.

The proposed system has several drawbacks. First, the MSRCR algorithm does not have adaptability towards different environments. Consequently, the algorithm parameters had to be adjusted carefully before use. This may limit the applications of the robot in ever-changing environments. Second, the detection and tracking algorithms used in the system are not sufficiently robust or precise for long-term robotic vision applications. Our future work will focus on high-level vision applications, including automatic navigation and object grabs. Additionally, advanced visual algorithms and tools including convolutional neural networks will be used to improve the designed robotic vision system.

## Figures and Tables

**Table 1:** Comparison of detection and tracking systems for underwater or amphibious applications.

Vision System	Hardware Platform	Image Size	Maximum Frame Rate	Working Scenarios
Proposed System	SoC	320 × 240	56.3 fps	Static and dynamic background
Shiau [19] et al.	PC	640 × 480	20.0 fps	Static background
Chuang [15] et al.	PC	2048 × 2048	5.0 fps	Dark environment
Lei [41] et al.	PC	352 × 288	3.3 fps	Swimming pool
Walther [42] et al.	PC	720 × 480	30.0 fps	Dark environment

**Table 2:** Experimental results of the visual detection subsystem.

Sequences	*PWC* (Proposed)	*Pr* (Proposed)	*PWC* (GBM)	*Pr* (GBM)
Sequences 1	0.018	0.821	0.092	0.733
Sequences 2	0.069	0.675	0.183	0.484
Sequences 3	0.030	0.985	0.052	0.924
Sequences 4	0.254	0.784	0.382	0.564

**Table 3:** Experimental results of the visual tracking subsystem.

Algorithm	Criteria	Sequence 1	Sequence 2	Sequence 3	Sequence 4
Proposed	*SR* (*CLE*)	100 (11.7)	91.8 (21.2)	100 (17.8)	100 (6.6)
CT	*SR* (*CLE*)	98.8 (13.8)	87.1 (27.1)	71.3 (27.1)	100 (8.6)
WMIL	*SR* (*CLE*)	100 (12.2)	77.3 (29.3)	98.7 (20.1)	92.1 (12.8)
HOG-SVM	*SR* (*CLE*)	100 (6.5)	85.1 (27.4)	100 (18.7)	100 (3.7)
TemplateMatch	*SR* (*CLE*)	100 (6.1)	84.7 (28.1)	80.3 (23.1)	100 (9.8)
MeanShift	*SR* (*CLE*)	10.3 (58.1)	14.3 (53.2)	35.4 (62.3)	100 (9.8)
VarianceRatio	*SR* (*CLE*)	12.2 (59.2)	50.6 (33.2)	56.3 (36.7)	100 (9.7)
PeakDifference	*SR* (*CLE*)	12.0 (58.9)	72.1 (31.7)	16.3 (67.2)	100 (7.2)
RatioShift	*SR* (*CLE*)	11.9 (45.6)	67.4 (28.2)	3.2 (87.3)	100 (8.4)
